# Adjusting for treatment switching in the METRIC study shows further improved overall survival with trametinib compared with chemotherapy

**DOI:** 10.1002/cam4.643

**Published:** 2016-01-27

**Authors:** Nicholas R. Latimer, Helen Bell, Keith R. Abrams, Mayur M. Amonkar, Michelle Casey

**Affiliations:** ^1^ School of Health and Related ResearchUniversity of SheffieldSheffieldUnited Kingdom; ^2^ Department of Health SciencesUniversity of LeicesterLeicesterUnited Kingdom; ^3^ Novartis Pharmaceuticals CorporationEast HanoverNew Jersey; ^4^ PfizerCollegevillePennsylvania

**Keywords:** BRAF protein human, clinical trial, drug therapy, melanoma, trametinib

## Abstract

Trametinib, a selective inhibitor of mitogen‐activated protein kinase kinase 1 (MEK1) and MEK2, significantly improves progression‐free survival compared with chemotherapy in patients with *BRAF* V600E/K mutation–positive advanced or metastatic melanoma (MM). However, the pivotal clinical trial permitted randomized chemotherapy control group patients to switch to trametinib after disease progression, which confounded estimates of the overall survival (OS) advantage of trametinib. Our purpose was to estimate the switching‐adjusted treatment effect of trametinib for OS and assess the suitability of each adjustment method in the primary efficacy population. Of the patients randomized to chemotherapy, 67.4% switched to trametinib. We applied the rank‐preserving structural failure time model, inverse probability of censoring weights, and a two‐stage accelerated failure time model to obtain estimates of the relative treatment effect adjusted for switching. The intent‐to‐treat (ITT) analysis estimated a 28% reduction in the hazard of death with trametinib treatment (hazard ratio [HR], 0.72; 95% CI, 0.52–0.98) for patients in the primary efficacy population (data cut May 20, 2013). Adjustment analyses deemed plausible provided OS HR point estimates ranging from 0.48 to 0.53. Similar reductions in the HR were estimated for the first‐line metastatic subgroup. Treatment with trametinib, compared with chemotherapy, significantly reduced the risk of death and risk of disease progression in patients with *BRAF* V600E/K mutation–positive advanced melanoma or MM. Adjusting for switching resulted in lower HRs than those obtained from standard ITT analyses. However, CI are wide and results are sensitive to the assumptions associated with each adjustment method.

## Introduction

Treatments inhibiting the enzymatic activity of mutated BRAF protein, a serine‐threonine protein kinase, in patients with advanced or metastatic melanoma (MM) became available in 2010 and have demonstrated improved progression‐free survival (PFS) and overall survival (OS) for patients with *BRAF‐*mutated melanoma 1, 2. *BRAF* mutations are present in 50% of patients with advanced melanoma 3, 4, 5. Trametinib is a mitogen‐activated protein kinase/extracellular signal–regulated kinase kinase (MEK) inhibitor that was approved in May 2013 in the United States.

METRIC (MEK Versus Dacarbazine [DTIC] or Paclitaxel [Taxol] in Metastatic Melanoma) was a randomized, multicenter phase 3 trial evaluating the efficacy and safety of trametinib compared with standard chemotherapy (dacarbazine or paclitaxel) in patients with advanced or metastatic (stage IIIc or IV) *BRAF* V600E/K mutation–positive melanoma. The prespecified number of PFS events was reached in October 2011. An intent‐to‐treat (ITT) analysis (comparing groups as randomized, without adjustment for treatment switching), conducted in February 2012, estimated a 58% reduction in the hazard for progression with trametinib (hazard ratio [HR], 0.42; 95% CI, 0.29–0.59) 2. The HR for death was 0.54 (95% CI, 0.32–0.92), but median OS had not been reached. In addition, the trial protocol allowed patients randomized to the chemotherapy control group who had progressive disease (PD) to switch onto trametinib and 51 patients (47.2%) had done so. Following this analysis, a protocol amendment dictated that immediate switching was permitted in patients randomized to the control group.

**Figure 1:**
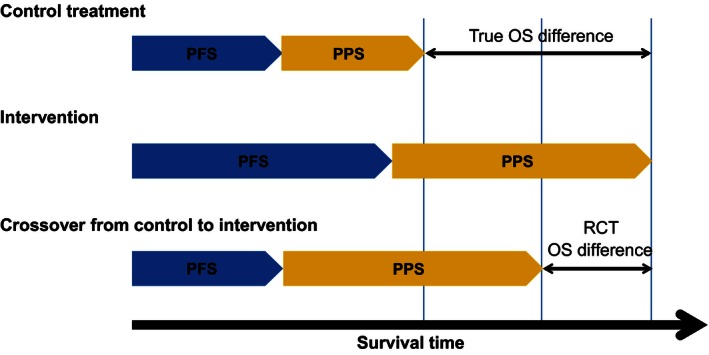
Treatment switching bias. OS, overall survival; PFS, progression‐free survival; PPS, postprogression survival; RCT, randomized controlled trial. (Reproduced with permission from Latimer et al. 6 .

When treatment switching is permitted, an ITT analysis can be confounded. If switching is permitted after PD, postprogression survival (PPS) in switching patients is likely to be extended compared with the PPS that would have been observed in the absence of switching. Therefore, an ITT analysis is likely to underestimate the OS effect of a novel treatment (Fig. 1) 6, 7. Accurate estimates of OS are important for patients, clinicians, and regulators, but are particularly crucial for health technology assessment because a lifetime horizon is generally taken in economic evaluations of interventions that affect survival 6, 8, 9, 10, 11. Inaccurate estimates of the OS advantage of a new treatment will result in inaccurate cost‐effectiveness results, possibly leading to inappropriate reimbursement decisions. This has serious implications for patients because access to effective treatments may be denied. The result is likely to be lost lives, lost quality of life, and an inefficient allocation of scarce healthcare budgets 12.

Statistical methods that adjust for treatment switching are available. However, naive “per‐protocol” methods that simply exclude switchers from the analysis, or censor them at the time of switch, will produce biased results because the propensity to switch is likely to be correlated with patient prognosis 6, 7, 13. Thus, more complex methods are required to improve upon the ITT analysis and account for treatment switching. Rank‐preserving structural failure time models (RPSFTM) and inverse probability of censoring weights (IPCW) are well‐established methods that may be used for this purpose 7, 14, 15, 16, 17, 18. A simplified two‐stage method for adjusting for treatment switching has been recently suggested 6.

In this study, we apply RPSFTM, IPCW, and two‐stage methods to account for confounding associated with treatment switching in METRIC to obtain a more reliable estimate of the true OS treatment effect of trametinib compared with chemotherapy, using a May 2013 data cut. In line with recent methodological recommendations 6, we assessed the suitability of each adjustment method in the context of METRIC.

## Materials and Methods

### Patients

**Figure 2:**
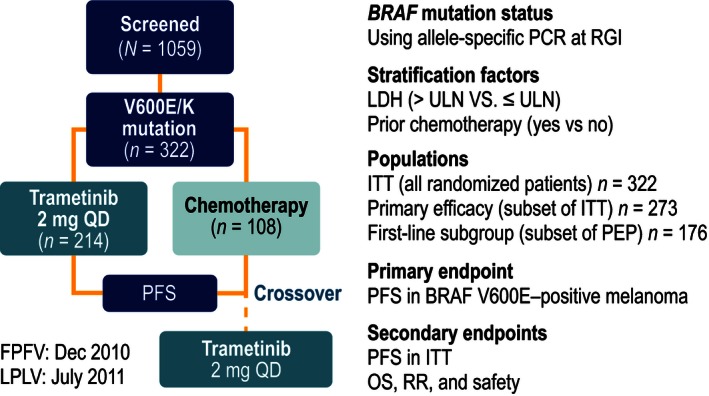
METRIC study design. FPFV, first patient, first visit; ITT, intent‐to‐treat; LDH, lactate dehydrogenase; LPLV, last patient, last visit; OS, overall survival; PCR, polymerase chain reaction; PEP, primary efficacy population; PFS, progression‐free survival; QD, once daily; RGI, Response Genetics, Inc; RR, response rate; ULN, upper limit of normal.

Patients enrolled on METRIC were randomized 2:1 to receive trametinib 2 mg once daily or chemotherapy (dacarbazine or paclitaxel). A total of 322 patients were enrolled on the study (Fig. 2). Patients were permitted to have previously received 1 line of chemotherapy treatment for advanced myeloma or MM. We conducted analyses for two groups: the primary efficacy population, including patients with *BRAF* V600E–positive MM with no history of brain metastases, and the subgroup within this population who received no previous treatment for advanced myeloma or MM. We focused on the primary efficacy population because an amendment to the METRIC trial protocol was made that dictated that the primary efficacy analysis was restricted to patients with the *BRAF* V600E mutation who did not have brain metastases, based upon data from the phase 2 study of trametinib, which indicated improved effectiveness in this group 2, 19. There were 273 patients in the primary efficacy population (trametinib, *n* = 178; chemotherapy, *n* = 95) and 176 patients in the first‐line metastatic subgroup (trametinib, *n* = 114; chemotherapy, *n* = 62). Further details of the study design are provided elsewhere 2, 20. The study cutoff date for this analysis of OS was May 20, 2013.

### Ethics

The study was approved by the institutional review board, and all patients provided written, informed consent to participate in the study.

### Statistical analyses

The RPSFTM estimates counterfactual survival times (i.e., survival times that would have been observed in the absence of treatment switching) 14. We fitted a Cox proportional hazards model (stratified for lactate dehydrogenase [LDH] level and prior chemotherapy for advanced or metastatic disease) to observed trametinib group survival times and counterfactual chemotherapy group survival times and estimated what the HR treatment effect would have been if switching had not occurred. The one‐parameter RPSFTM relies on the assumptions that the treatment effect is equal across all patients, relative to the duration of time the treatment was taken for (the “common treatment effect” assumption), and, in the absence of treatment, that survival times are independent of the randomized group (the “randomization” assumption). We assessed the performance of the RPSFTM by comparing counterfactual survival times estimated for the control group with those estimated for the experimental group (i.e., survival times that would have been observed if no patients in either group received treatment). If this provides an HR close to 1, this signals that the model's estimation procedure has performed well. However, this does not mean that the assumptions associated with the method are justified, or that the data fit the model. To provide a further check on the suitability of the RPSFTM we visually compared the complete counterfactual survival curves. We also used the switching‐specific treatment effect obtained from the two‐stage estimation method (described below) to assess the plausibility of the common treatment effect assumption.

The Iterative Parametric Estimation (IPE) adjustment method adapts the RPSFTM by using a parametric estimation procedure 21. We applied the IPE method in this case study, but found that due to convergence issues (i.e., treatment effects not being identified by the iterative estimation procedure) it was consistently outperformed by the RPSFTM. Therefore, we do not report on this method further.

The IPCW method artificially censors patients at the point of treatment switch and estimates weights for the observations associated with remaining patients according to their baseline and time‐varying demographic and disease‐related characteristics to adjust for any potential confounding created by the artificial censoring 15, 22. These weights are then used in a weighted Cox proportional hazards regression model to obtain an adjusted estimate of the treatment effect 15, 22. The method is reliant on the assumption that the model includes information on all prognostic characteristics (the “no unmeasured confounders” assumption). To address the validity of our IPCW analysis, we considered the plausibility of the no unmeasured confounders assumption given the data collected in METRIC.

The two‐stage estimation method can be applied when switching occurs after a disease‐related time point 6. For METRIC, we used the PD time point as a secondary baseline for all control group patients and compared PPS in control group switchers and nonswitchers using an accelerated failure time model, adjusting for prognostic characteristics measured at baseline and PD. We used the acceleration factor (AF) obtained from this model to adjust survival times observed in switching patients to obtain counterfactual survival times. We then fitted a Cox proportional hazards regression model (stratified for LDH level and prior chemotherapy for advanced or metastatic disease) to the observed trametinib group survival times and the counterfactual chemotherapy group survival times to estimate a treatment switching‐adjusted HR. This method is theoretically inferior to the IPCW method if switching does not occur immediately upon PD because it does not adjust for time‐dependent confounding that may occur between PD and switching. However, it has been shown to be less prone to convergence issues and bias than the IPCW method in scenarios when sample sizes are small and switching proportions are high, and, unlike the RPSFTM method, does not make the common treatment effect assumption 6, 23, 24. To assess the validity of the two‐stage analysis, we examined the time between PD and switching to investigate potential bias from time‐dependent confounding.

Censoring in counterfactual datasets can be problematic because the treatment received affects the probability that the survival time of an individual is censored by the study end date 23. Possible bias from this can be avoided by breaking the dependence between censoring time and treatment by “recensoring” at an earlier time point based upon the observed administrative censoring time and the size of the treatment effect 25, 26. A drawback to recensoring is that it involves “throwing away” information; if the treatment effect changes over time, recensoring will lead to a biased estimate of the average treatment effect because longer term data are discarded. To address this, we conducted our RPSFTM and two‐stage analyses with and without recensoring.

We used Stata version 13.1 software 27 to carry out all of our analyses, and we used the strbee command to apply the RPSFTM method 28. Further details on the implementation of each method, including information on baseline and time‐dependent characteristics incorporated within the IPCW and two‐stage analyses, are presented in the Data S1.

## Results

**Figure 3:**
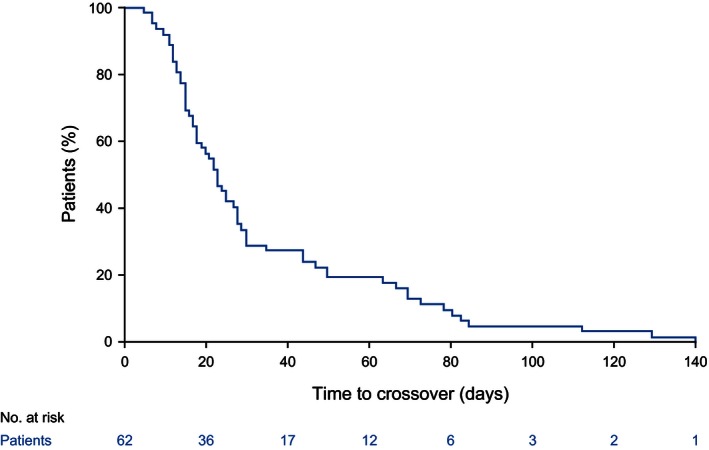
Time to switch from progression for patients on the chemotherapy control arm of the METRIC study who switched to trametinib treatment.

In the primary efficacy population, 64 patients (67.4%) in the control group had switched to trametinib at the time of data cutoff. Of these, 62 patients switched after disease progression had occurred – hence, we deemed it unnecessary to attempt to adjust for the effect of switching on estimates of the treatment effect on PFS, but it is clear that control group OS is likely to be confounded. There were 109 deaths (61.2%) in the trametinib group and 61 (64.2%) in the control group (42 switchers and 19 nonswitchers). Figure 3 presents the time from PD until switch in switching patients. Approximately 75% of switching occurred fewer than 40 days after PD, with a median time from progression until switching of 23 days.

The ITT analysis for the primary efficacy population showed that OS was improved for patients randomized to trametinib compared to those randomized to chemotherapy (HR from a Cox proportional hazards regression model stratified for LDH level and prior chemotherapy for advanced or metastatic disease, 0.72; 95% CI, 0.52–0.98).

**Figure 4:**
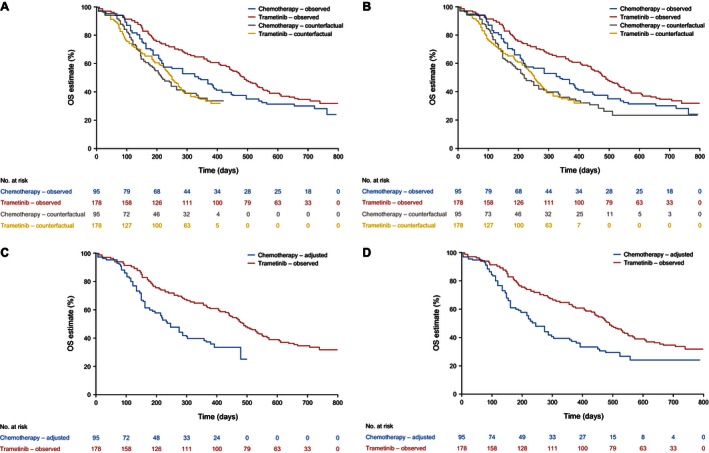
Overall survival in primary efficacy population. (A). Rank‐preserving structural failure time models (RPSFTM) with recensoring. (B). RPSFTM without recensoring. (C). Two‐stage method with recensoring. (D). Two‐stage method without recensoring.

**Figure 5:**
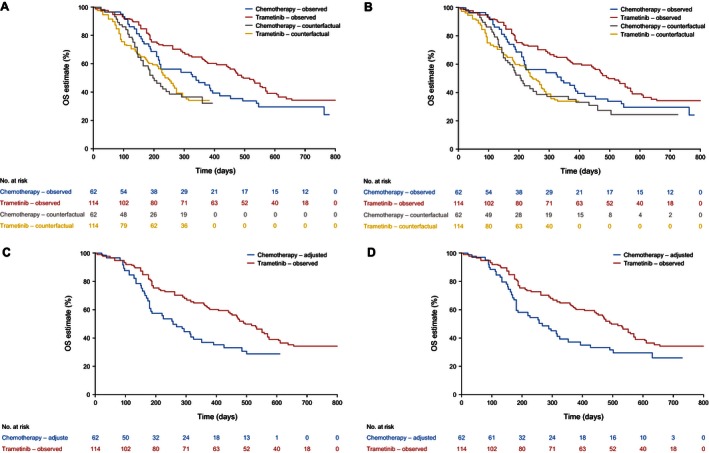
Overall survival in first‐line metastatic primary efficacy population. (A). Rank‐preserving structural failure time models (RPSFTM) with recensoring. (B). RPSFTM without recensoring. (C). Two‐stage method with recensoring. (D). Two‐stage method without recensoring.

The results of the adjustment analyses are presented in Table 1, and Figure 4 and Figure 5 illustrate Kaplan–Meier curves representing observed survival times for the trametinib group and counterfactual survival times estimated for the chemotherapy group by the RPSFTM and two‐stage adjustment methods. The analyses that adjusted for treatment switching resulted in reduced HRs and a larger divergence between Kaplan–Meier curves for the trametinib and chemotherapy groups. The RPSFTM method produced an OS HR of 0.38 (95% CI, 0.15–0.95) when recensoring was incorporated and 0.49 (95% CI, 0.25–0.96) when recensoring was not undertaken. The IPCW estimate of the OS HR was 0.48 (95% CI, 0.25–0.91), and the two‐stage method provided an OS HR of 0.43 (95% CI, 0.20–0.96) when recensoring was incorporated and 0.53 (95% CI, 0.29–0.97) when recensoring was not undertaken (Table 1).

**Table 1:** RPSFTM, IPCW and two‐stage estimates of overall survival treatment effect

Description	HR			CF HR comparison^a^	Median (chemo group, days)
	Point estimate	Lower 95% CI	Upper 95% CI		
METRIC primary efficacy population					
ITT analysis	0.72	0.52	0.98	–	338.0
RPSFTM	0.38	0.15	0.95	1.00	220.0
RPSFTM without recensoring	0.49	0.25	0.96	1.00	220.0
IPCW^b^	0.48	0.25	0.91	–	–
Two‐stage method	0.43	0.20	0.96	–	244.2
Two‐stage method without recensoring	0.53	0.29	0.97	–	244.2
METRIC first‐line metastatic primary efficacy population					
ITT analysis	0.67	0.45	1.00	–	338.0
RPSFTM	0.33	0.11	1.00	1.00	196.0
RPSFTM without recensoring	0.44	0.20	1.00	1.00	207.1
IPCW^b^	0.33	0.16	0.68	–	–
Two‐stage method^b^	0.51	0.26	1.00	–	256.5
Two‐stage method without recensoring^b^	0.55	0.30	1.00	–	268.5

CI, confidence interval; HR, hazard ratio; IPCW, inverse probability of censoring weights; ITT, intent‐to‐treat population; LDH, lactate dehydrogenase; OS, overall survival; RPSFTM, rank‐preserving structural failure time models.

The ITT HRs presented in the table are estimated using Cox PH models, stratified for LDH. GSK use a Pike estimator to calculate ITT HRs. This resulted in a HR of 0.72 (95% CI, 0.52–1.01) for the Primary Efficacy Population and a HR of 0.74 (95% CI, 0.49–1.12) for the first‐line metastatic subpopulation. Results are given to 2 decimal places.

a CF HR comparison represents the comparison of counterfactual survival times in each randomized group if no patients in either group received any treatment, given the treatment effect estimated by the method. Successful estimation would result in a CF HR of 1.00.

b Analyses did not fully converge when all covariates were included. Results presented represent those from most complete application that converged (see Data S1. for full details).

The first‐line metastatic subgroup included 176 patients (trametinib, *n* = 114; chemotherapy, *n* = 62). In this subgroup, 43 patients on chemotherapy (69.4%) switched to trametinib. There were 70 deaths (61.4%) in the trametinib group and 40 (64.5%) in the chemotherapy group (29 switchers and 11 nonswitchers). The first‐line subgroup ITT analysis indicated that the treatment benefit of trametinib compared with chemotherapy was slightly greater than that for the entire primary efficacy population (HR from a Cox proportional hazards regression model stratified for LDH level, 0.67; 95% CI, 0.45–1.00), although the difference was not statistically significant. Switching adjustment methods again produced reduced HRs and a larger divergence between the Kaplan–Meier curves for the trametinib and chemotherapy groups (Fig. 5). The RPSFTM method produced an OS HR of 0.33 (95% CI, 0.11–1.00) when recensoring was incorporated and 0.44 (95% CI, 0.20–1.00) when recensoring was not undertaken. The IPCW estimate of the OS HR was 0.33 (95% CI, 0.16–0.68). The two‐stage method provided an OS HR of 0.51 (95% CI, 0.26–1.00) when recensoring was incorporated and 0.55 (95% CI, 0.30–1.00) when recensoring was not undertaken (Table 1).

## Discussion

Trametinib was previously shown to improve PFS and OS compared with chemotherapy in patients with *BRAF* V600E/K–mutant melanoma 2. In the updated ITT analysis using the May 2013 data cut, the OS treatment effect was reduced and of borderline statistical significance, potentially due to the confounding effects of switching.

After adjustment for treatment switching, trametinib reduced the risk of death compared with chemotherapy, with HRs substantially lower than those from the ITT analysis, which estimated a 28% reduction in the hazard of death with trametinib. The most plausible RPSFTM, IPCW, and two‐stage method analyses estimated reductions of between 47% and 52%. CI were wide and of borderline statistical significance, largely due to the design of the adjustment methods. The RPSFTM and two‐stage methods retained the *P* value from the ITT analysis by design (though bootstrapping could alternatively be undertaken), thus, in situations when the point estimate of the HR is reduced, CI widen. The IPCW analysis does not retain the ITT analysis *P* value (*P* = 0.04 in the ITT analysis reduced to *P* *= *0.02 in the IPCW analysis).

The point estimate reductions in the OS HR were not unexpected; the majority of patients randomized to the chemotherapy group switched to trametinib (64 of 95 patients [67.4%]). Given the large PFS treatment effect associated with trametinib, it is reasonable to expect that switching patients will live longer than they would have if they had not switched treatments. Adjusting for the treatment switching observed in the majority of chemotherapy group patients would therefore be expected to have a substantial impact on the estimate of the OS treatment effect. Although adjustment methods such as the RPSFTM, IPCW, and two‐stage estimation are likely to produce smaller bias than naive per‐protocol adjustments 6, 7, 13, each method has important assumptions that must be considered.

The RPSFTM is reliant upon the common treatment effect assumption. This assumption may be implausible given that most switching patients received trametinib after PD, potentially resulting in a diminished capacity to benefit compared to patients who received trametinib immediately upon randomization. The comparison of counterfactual survival times estimated for the control group and the experimental group resulted in HRs of 1.00, suggesting that the RPSFTM analyses had worked well. However, a visual inspection of the counterfactual survival curves presented in Figure 4 A and B and Figure 5 A and B suggests that the fit of the data to the model was not perfect, which may place some doubt on the validity of the common treatment effect assumption. Although the common treatment effect assumption is impossible to test with precision, the two‐stage method provides an estimate of the postprogression treatment effect of trametinib specifically in switching patients compared with patients who did not switch, and of the OS treatment effect of trametinib compared to chemotherapy adjusted for switching. If these estimated effects are similar, the common treatment effect assumption may be made more confidently, because it would appear that patients are receiving a similar benefit from the experimental treatment no matter when they received it. For the primary efficacy population analysis, the two‐stage method provided an estimate of the postprogression AF associated with switching to trametinib of 1.65 (95% CI, 1.11–2.45; an AF > 1 indicates a treatment that extends survival time). An OS AF of 1.75 was estimated for the trametinib group compared with the chemotherapy group, once adjustments had been made for switching. Hence, although the two‐stage analysis is prone to some bias, there appeared to be no strong evidence against the common treatment effect assumption.

The IPCW is reliant on the no unmeasured confounders assumption. Results are prone to substantial error with small sample sizes and large switching proportions 6, 23. The large amount of baseline and time‐dependent data available on important prognostic variables collected during the METRIC trial lends itself to an IPCW analysis (see Data S1.for full details on prognostic factors included in the IPCW and two‐stage analyses). However, the small sample size means that the IPCW results must be interpreted with caution: only 31 control group patients did not switch to trametinib, and these form the basis of the IPCW control group survival estimates. This is particularly relevant for the first‐line subgroup analyses, for which the sample size is further reduced (only 19 control group patients did not switch). Indeed, weighting models did not successfully converge when all covariates were included bringing the feasibility of the no unmeasured confounders assumption into question. Health‐related quality of life scores based upon the EuroQol 5D (EQ‐5D) were excluded from the primary efficacy population analysis, and European Organisation for Research and Treatment of Cancer quality of life 30 (EORTC QLQ‐30) domains were excluded from the first‐line subgroup analysis (see Data S1. for further details). However, in each case the alternative quality of life measure was retained in the analysis (i.e., EQ‐5D was retained when EORTC QLQ‐30 was excluded, and vice‐versa) and therefore these exclusions may not represent important violations of the no unmeasured confounders assumption.

The two‐stage estimation method is reliant upon the no unmeasured confounders assumption at PD and assumes that no additional time‐dependent confounding occurs between PD and the time of treatment switch. For the primary efficacy population analysis, models converged that incorporated all covariates; therefore, the no unmeasured confounders assumption is not unreasonable. However, in the first‐line subgroup, EORTC QLQ‐30 covariates were excluded for model convergence to be achieved; therefore, the results are less robust (although EQ‐5D covariates were retained, and hence the no unmeasured confounders assumption may remain reasonable). Although Figure 3 suggests fairly rapid switching after PD (median 23 days), some patients took much longer to switch. Three patients switched more than 100 days after PD, leaving scope for additional time‐dependent confounding to occur. Although the two‐stage method cannot be expected to produce completely unbiased estimates, remaining bias may be small because most switching happened soon after PD.

Recensoring was a key issue in our METRIC analysis. Whilst the issues around recensoring have been previously considered 6, 25, 29, 30, we are not aware of any previous analyses that have presented adjustment analysis results with and without incorporating recensoring. Figures 4 and 5 show that for the primary efficacy population, recensoring led to a maximum survival time of 422 days (14.1 months) for the RPSFTM and 498 days (16.6 months) for the two‐stage method, compared with a maximum observed survival time of 792 days (26.4 months) in METRIC. It is noticeable that the largest gap between the observed trametinib and chemotherapy Kaplan–Meier curves occurs at approximately 400 days (13.0 months), after which the survival curves begin to converge. This may be partly due to switching. However, only 14 of the 64 switchers remained on trametinib after this time point; therefore, it seems likely that most of the impact of switching would already be apparent at 422 days. Despite this, we found that artificially censoring all patients in both the chemotherapy and trametinib groups at 422 days resulted in an ITT HR of 0.56, compared with the ITT HR of 0.72 at the end of follow‐up. We hypothesize that at least part of the convergence of the survival curves after 422 days is associated with a reduction in the trametinib treatment effect over time; therefore, recensoring at early time points will lead to an overestimate of the true longer term average OS treatment effect. For this reason, we believe that RPSFTM and two‐stage analyses without recensoring provide more credible results for the primary efficacy population. For the first‐line subgroup, the impact of recensoring is different for the RPSFTM and two‐stage analyses. For the RPSFTM, recensoring led to a maximum survival time of 392 days (12.9 months) compared to 611 days (20.1 months) for the two‐stage analysis (Fig. 5). Hence the loss of information associated with recensoring is much less pronounced for the two‐stage analysis. Artificially censoring all patients at 611 days resulted in an ITT HR of 0.66 for the first‐line subgroup, compared to 0.67 at the end of follow‐up. Therefore, in the first‐line subgroup our preference is for the RPSFTM analysis without recensoring and the two‐stage analysis with recensoring.

These analyses that adjust for the switching observed in METRIC confirm that trametinib improved OS, compared with chemotherapy, in patients with MM with a V600E/K *BRAF* mutation. The size of the treatment effect was considerably larger than that estimated using a standard, unadjusted ITT analysis. Although adjustment methods have important limitations, they are likely to provide a more reliable estimate of the true treatment effect of trametinib than an ITT analysis. Given the particular characteristics of METRIC, the RPSFTM, and two‐stage methods that do not incorporate recensoring may be more appropriate than those that do. A key advantage of the two‐stage and IPCW analyses is that they do not require the common treatment effect assumption, although this assumption appears reasonable in this case. Two‐stage analyses are potentially more prone to bias than IPCW analyses because they do not account for all time‐dependent confounding, but it was possible to incorporate more covariates in the two‐stage models than the IPCW models in the METRIC analysis; hence, the IPCW may be more prone to unmeasured confounding. In particular, for the first‐line treatment subgroup, we believe that the IPCW provided implausible results – the estimated HR appears unrealistically low. The IPCW method is prone to error in scenarios similar to those exhibited by the first‐line subgroup within the METRIC trial – with very low numbers of control group patients not switching and potential unmeasured confounding 6, 23. With the exception of this analysis, the complex adjustment methods provided reassuringly similar estimates of the true treatment effect of trametinib compared with chemotherapy on OS, suggesting that the hazard of death is reduced by 47–52% in the primary efficacy population and 49–56% in the first‐line subgroup, compared with ITT estimates of 28% and 33% in the primary efficacy population and first‐line subgroups, respectively. Clinically, these results suggest that the expected OS benefit associated with trametinib is substantially greater than that previously estimated using standard ITT analyses.

## Conflict of Interest

Employment: M. Amonkar, GlaxoSmithKline (at time of analysis), Novartis (at time of submission); M. Casey, GlaxoSmithKline (at time of analysis), Pfizer (at time of submission). Stock or Other Ownership: M. Amonkar, GlaxoSmithKline, Novartis; M. Casey, GlaxoSmithKline, Pfizer. Honoraria: N. Latimer, Sanofi, Astellas, Bayer, Pfizer, AstraZeneca; H. Bell, Pfizer. Consulting or Advisory Role: N. Latimer, Sanofi, Astellas, Bayer, Pfizer, AstraZeneca, GlaxoSmithKline, Novartis, Bristol‐Myers Squibb; K. Abrams, Amaris, Astellas, Allergan, AstraZeneca, Bayer, Bristol‐Myers Squibb, Creativ‐Ceutical, GlaxoSmithKline, Janssen, Merck, Novartis, Novo Nordisk, OptumInsight, Oxford Outcomes, PRMA Consulting, Roche, RTI International. Research Funding: N. Latimer, GlaxoSmithKline, Novartis, Eisai, BMS; H. Bell, GlaxoSmithKline, Novartis, Eisai; K. Abrams, Pfizer.

## Supporting information


**Data S1.** Implementation of adjustment methods.
