# Supplementary material for: Adjusting for treatment switching in the METRIC study shows further improved overall survival with trametinib compared with chemotherapy
Source: Cancer Med. 2016 Jan 27;5(5):806–15. doi: 10.1002/cam4.643 (PMC4864810; doi:10.1002/cam4.643)
Supplement: Supplementary file 1 — Data S1. Implementation of adjustment methods. [file CAM4-5-806-s001.docx]

**SUPPORTING INFORMATION**

***Implementation of the RPSFTM method***

The standard 1-parameter rank-preserving structural failure time model (RPSFTM) splits the observed event time (*T_i_*) for each patient into 2, time spent off treatment (*T_Ai_*), and time spent on treatment (*T_Bi_*). For patients who are randomized to the intervention treatment, and who do not switch onto the control treatment (that is, when compliance is full in the treatment group), *T_Ai_* is equal to 0. For patients randomized to the control group who do not switch onto the intervention (eg, compliance is full in the control group) *T_Bi_* is equal to 0. However, for patients who switch treatments (for whom compliance is imperfect) both *T_Ai_* and *T_Bi_* will be > 0.

The standard 1-parameter RPSFTM method relates *T_i_* to the counterfactual event time (*U_i_*) with the following causal model:

$U_{i}=T_{A_{i}}+e^{\psi0}T_{B_{i}}$ (1)

Here $e^{-\psi0}$ represents the acceleration factor (AF) associated with the intervention. By defining a binary process $X_{i}(t)$ which equals 1 when a patient is on the intervention treatment, and equals 0 when the patient is on control treatment, the causal model can be rewritten as:

$U_{i}=\int_{0}^{T_{i}} \exp\left[ \psi X_{i}(t) \right]\mathrm{dt}$ (2)

The value of *ψ* is estimated using g-estimation: *U_i_* is estimated using the causal model for a range of potential values of *ψ*, and the true value of *ψ* is that for which *U(ψ)* is independent of randomized groups, based upon a g-test. As described by Mark and Robins (1993), a log-rank or Wilcoxon test can be used for the RPSFTM g-test in a non-parametric setting, testing the hypothesis that the baseline (untreated) survival curves are identical in the 2 treatment groups, or a Wald test could be used for parametric models.^1^ The point estimate of *ψ* is that for which the test (z) statistic equals 0.

There are alternative ways in which the RPSFTM method can be implemented.^2^ We define these approaches as follows:

1. ‘On treatment’ analysis. This involves a direct implementation of the RPSFTM model shown in equation.^1^ Under an ‘on treatment’ approach it is assumed that a treatment benefit is only received while treatment is being received – any treatment effect immediately disappears upon treatment discontinuation. This results in a ‘causal’ treatment effect being estimated for the experimental treatment. It is important to note that this does not provide an estimate of the effect of being randomized to the treatment group, because it essentially provides an estimate of the effect of ‘full’ treatment (with no discontinuation) compared to no treatment. In reality, treatment discontinuation is likely: patients do not receive treatment forever. In the context of an economic evaluation it may be argued that what is required from the analysis of the trial is an estimate of the treatment effect associated with being in the intervention group compared to the control group, rather than the causal treatment effect measured only while treatment is being received. This can be obtained from an ‘on treatment’ RPSFTM analysis: first the causal treatment effect is estimated, and corresponding counterfactual survival times are estimated for the control group. Then, the survival times observed in the experimental group (incorporating any treatment discontinuation) are compared to the control group counterfactual survival times to estimate the adjusted treatment effect.
2. ‘Treatment group’ analysis. The RPSFTM method can also be applied on a ‘treatment group’ basis. If a treatment effect that continues beyond treatment discontinuation is expected the RPSFTM method could be applied assuming a lagged treatment effect, or on a ‘treatment group’ basis – where patients in the experimental group are always considered to be ‘on’ treatment and patients that switch remain ‘on’ treatment from the time of switch until death. This analysis ignores treatment discontinuation times and estimates the effect associated with being randomized to the experimental group, rather than the ‘causal’ effect received while taking the experimental treatment. In this sense, this approach is more similar to a standard intention-to-treat (ITT) analysis of randomized groups.

A key limitation associated with the RPSFTM method is that in its simple 2-exposure (‘on’ or ‘off’ treatment) form, patients are always assumed to be either ‘on’ or ‘off’ treatment. In circumstances where the control treatment is active this may cause problems with the analysis. A multi-parameter version of the model could be used in order to incorporate multiple exposures, but analysts have found that this leads to a lack of power and meaningful point estimates of causal effects are not obtained.^1,3-7^

Limited to a model with 2 exposures, an ‘on treatment’ RPSFTM or iterative parameter estimation (IPE) analysis assumes that any period of time not spent ‘on’ the experimental treatment is spent ‘off’ treatment, and any treatments taken during these periods are effectively combined and considered as similar. Where the comparator arm of the trial is placebo, or a best supportive or palliative care, the 2-exposure RPSFTM is reasonable. However if the comparator is an active treatment, and if post-study therapies are likely to be non-active, the 2-exposure ‘on treatment’ RPSFTM model may not be clinically plausible. Combining the active comparator and the non-active post-study therapies into 1 ‘off’ group may lead to a dilution of the effectiveness associated with the comparator and an over-estimate of the treatment effect.

In these circumstances it may be more reasonable to use a ‘treatment group’ analysis in order to estimate an approximate treatment effect for the entire treatment sequence received by the experimental group compared to the sequence received by the control group (except this is adjusted to account for treatment switching). This would require the assumption that when a control group patient switches treatment, he/she switches onto the experimental group treatment sequence – it is assumed that the treatment sequence received by a switching patient is similar to that received by a patient initially randomized to the experimental group, and that the relative effect of that sequence is similar to that received by experimental group patients. Hence the ‘common treatment effect’ assumption refers to the set of treatments received after switch, rather than simply to the experimental treatment alone.

In a ‘treatment group’ analysis using a simple 2-exposure RPSFTM groups still have to be combined – time spent ‘on’ treatment is any time after experimental treatment initiation, ignoring subsequent treatment discontinuation; and time spent ‘off’ treatment is all time prior to experimental treatment initiation, including time after control group treatment discontinuation if switching does not occur. Hence such analyses may dilute the effectiveness associated with the experimental treatment and the active comparator treatment as for both groups time spent after discontinuation of the treatments is attributed to those treatments. However, while not providing a causal effect of the novel treatment, this approach may provide a reasonable estimate of the treatment effect of being randomized to the experimental treatment group rather than the control group. Rather than diluting only the comparator group, as would be the case in an ‘on treatment’ analysis, the ‘treatment group’ approach dilutes both groups and may be preferable in situations where the comparator treatment is active and importantly different to post-study treatments, where post-study treatments are similar and have similar effectiveness in switching patients and patients initially randomized to the experimental group, and particularly where some of the benefit associated with the experimental treatment is maintained beyond treatment discontinuation. The ‘treatment group’ approach requires that the post-study treatments received in the randomized groups are representative of a realistic treatment pathway – in which case if subsequent benefits received due to post-study therapy differ depending upon previous treatment these benefits can appropriately be attributed to the experimental treatment group from the perspective of a pragmatic health technology assessment or economic evaluation.

In the METRIC (MEK Versus Dacarbazine [DTIC] or Paclitaxel [Taxol] in Metastatic Melanoma) trial the chemotherapy comparator is active, and post-study treatments were available. We applied the RPSFTM on ‘treatment group’ and ‘on treatment’ bases, and obtained similar results – suggesting that in this instance the method of application made little difference to results. Owing to the active nature of the comparator, we present results from the RPSFTM ‘treatment group’ analysis.

We used the strbee Stata program to apply the RPSFTM ‘treatment group’ method.^8^ The analysis consisted of 2 steps. Step 1 involved estimating the treatment effect *ψ*, using g-estimation. We used a log-rank test to determine the value of *ψ* for which counterfactual survival times were independent of randomized groups. Step 2 involved estimating the HR for overall survival for randomization to trametinib vs randomization to chemotherapy with no switching to trametinib by fitting a Cox proportional hazards regression model to the trametinib failure times as observed in the METRIC trial and re-censored adjusted failure times for chemotherapy patients based on the estimate of *ψ* produced by Step 1.

We assumed that a treatment benefit could be received even after treatment had been discontinued: the treatment indicator remained set to ‘1’ (ie, ‘on’ treatment) for all time periods after treatment was initiated. To investigate the potential impact of recensoring, once potentially appropriate applications of the RPSFTM/IPE methods had been identified we re-ran these without incorporating recensoring.

Confidence intervals around the RPSFTM adjusted treatment effect were calculated by retaining the *P*-value from the ITT analysis.^9^

***Implementation of the IPCW method***

We based our application of the inverse probability of censoring weights (IPCW) method on Stata code presented by Fewell *et al.*^10^ The IPCW method entails 3 main steps. Step 1 involves the creation of a panel dataset. For all patients, follow-up time from randomization until death or censoring is partitioned into intervals. Observations beyond the time of treatment switch are dropped for patients who switched, and an indicator for informative censoring is created to indicate that these patients were informatively censored at the time of switching. At the beginning of each interval, time-dependent variables that may be predictive of informative censoring or failure are calculated and updated.

Using the panel data created in Step 1, for each chemotherapy patient *i* and interval (*j*), stabilized weights, *SWi(j)*, were estimated, as presented in equation (3):^11^

$\hat{W}(t)=\prod_{k=0}^{t} \frac{\Pr\left[ C(k)=0|\bar{C}\left( k-1 \right)=0,\bar{A}\left( k-1 \right), V, T>k \right]}{\Pr\left[ C(k)=0|\bar{C}(k-1)=0,\bar{A}\left( k-1 \right),\bar{L}\left( k \right), T>k \right]}$ $\hat{W}(t)=\prod_{k=0}^{t} \frac{\Pr\left[ C(k)=0|\bar{C}\left( k-1 \right)=0,\bar{A}\left( k-1 \right), V, T>k \right]}{\Pr\left[ C(k)=0|\bar{C}(k-1)=0,\bar{A}\left( k-1 \right),\bar{L}\left( k \right), T>k \right]}$ (3)

Where $C(k)$ is an indicator function demonstrating whether or not informative censoring (switching) had occurred at the end of interval *k*, and $\bar{C}\left( k-1 \right)$ denotes censoring history up to the end of the previous interval *(k-1)*. $\bar{A}\left( k-1 \right)$ denotes an individual’s treatment history up until the end of the previous interval *(k-1)*, and *V* is an array of an individual’s baseline covariates. $\bar{L}\left( k \right)$ denotes the history of an individual’s time-dependent covariates measured at or prior to the beginning of interval *k*. Hence the numerator of (1) represents the probability of an individual remaining uncensored (not switched) at the end of interval *k* given that that individual was uncensored at the end of the previous interval *(k-1)*, conditional on baseline characteristics and past treatment history. The denominator represents that same probability conditional on baseline characteristics, time-dependent characteristics and past treatment history. When the cause of informative censoring is treatment switching, past treatment history is removed from the model because as soon as switching occurs the individual is censored.

The denominator of these weights is the probability of remaining uncensored (ie, not switching to trametinib) to the end of interval (*j*) given baseline and time-dependent covariates. The numerator of the weights is the probability of remaining uncensored (ie, not switching to trametinib) to the end of interval (*j*) given only baseline covariates. Estimates were obtained by fitting pooled logistic models with informative censoring (designating treatment switch) as the dependent variable.

For the numerator, the covariates included in the logistic models were:

- Age (continuous variable)
- Sex (0 = Male; 1 = Female)
- Receipt of prior chemotherapy for advanced or metastatic disease (0 = No; 1 = Yes)
- Receipt of prior immunotherapy (0 = No; 1 = Yes)
- Eastern Cooperative Oncology Group (ECOG) performance status code (0 = ECOG of 0; 1 = ECOG of ≥ 1)
- Lactate dehydrogenase level (0 = equal to or lower than upper normal limit; 1 = higher than upper normal limit)
- Disease stage at screening (0 = Stage III, IVM1a or IVM1b; 1 = IVM1c)
- Visceral disease at screening (0 = No; 1 = Yes)
- Number of disease sites (0 = ≥ 3 sites; 1 = < 3 sites)
- Sum of lesion diameters (continuous variable)
- EuroQol 5D (EQ-5D) utility score (continuous variable)
- European Organisation for Research and Treatment of Cancer quality of life 30 score (EORTC QLQ-30) physical, role, emotional, cognitive, social, fatigue, nausea and vomiting, pain, dyspnea, insomnia, appetite loss, constipation, diarrhea, financial difficulties, global health status domains) (continuous variables)

For the denominator, the covariates included in the logistic models were:

- Baseline: Age (continuous variable)
- Sex (0 = Male; 1 = Female)
- Baseline: Receipt of prior chemotherapy for advanced or metastatic disease (0 = No; 1 = Yes)
- Baseline: Receipt of prior immunotherapy (0 = No; 1 = Yes)
- Baseline: ECOG performance status code (0 = ECOG of 0; 1 = ECOG of ≥ 1)
- Baseline: Lactate dehydrogenase level (0 = equal to or lower than upper normal limit; 1 = higher than upper normal limit)
- Baseline: Disease stage at screening (0 = Stage III, IVM1a or IVM1b; 1 = IVM1c)
- Baseline: Visceral disease at screening (0 = No; 1 = Yes)
- Baseline: Number of disease sites (0 = ≥ 3 sites; 1 = < 3 sites)
- Baseline: Sum of lesion diameters (continuous variable)
- Baseline: EQ-5D utility score (continuous variable)
- Baseline: EORTC QLQ-30 (physical, role, emotional, cognitive, social, fatigue, nausea and vomiting, pain, dyspnea, insomnia, appetite loss, constipation, diarrhea, financial difficulties, global health status domains) (continuous variables)
- Time dependent: Time of progression (continuous variable)
- Time dependent: ECOG performance status code (0 = ECOG of 0; 1 = ECOG of ≥ 1)
- Time dependent: lactate dehydrogenase (LDH [0 = equal to or lower than upper normal limit; 1 = higher than upper normal limit])
- Time dependent: Best response on study treatment (0 = Not evaluable; 1 = Progressive disease; 2 = Stable disease; 3 = Partial response; 4 = Complete response)
- Time dependent: Sum of lesion diameters (continuous variable)
- Time dependent: Presence of new lesions (0 = No; 1 = equivocal; 2 = Yes)
- Time dependent: Serious adverse event experienced (0 = No; 1 = Yes)
- Time dependent: Serious adverse event current (0 = No; 1 = Yes)
- Time dependent: EQ-5D utility score (continuous variable)
- Time dependent: EORTC QLQ-30 (physical, role, emotional, cognitive, social, fatigue, nausea and vomiting, pain, dyspnea, insomnia, appetite loss, constipation, diarrhea, financial difficulties, global health status domains) (continuous variable)

Both the model for the numerator and the model for the denominator were only fit to patients in the control group, because patients in the experimental group were not at risk of switching. The probability of switching is set to 0 in the experimental group and observations on these patients are not used in the logistic models. The model for the denominator was only fit to patients in the control group after the time of disease progression because, according to the study protocol, treatment switching was not possible before disease progression. Thus the probability of switching is set to 0 (the probability of remaining uncensored due to switching is set to 1) for control group patients prior to disease progression in the denominator of the weight. This approach is not used for the numerator of the weight, because it involves the use of time-dependent information on the time of disease progression. In fact, 2 of the 64 primary efficacy population chemotherapy patients who switched onto trametinib did so before disease progression had been observed. For these patients, disease progression was censored at the last adequate assessment prior to initiation of the new treatment, which occurred 91 days and 9 days prior to the switching date for each patient respectively. To investigate the impact of this we ran 2 sets of IPCW analyses – in 1 set these patients were not included in the stabilized weight denominator risk set because disease progression had not been observed. In the second set we assumed that disease progression was observed at the time of switching, and thus these patients were included within the stabilized weight denominator risk set. We found that this led to only marginal differences in estimates of the treatment effect, and because this approach allowed more observations to be included within the logistic models, we present results from this set of analyses in our manuscript.

We used Stata’s rcsgen command to generate restricted cubic splines for a time-dependent intercept that was used in the numerator and denominator, and tested models that incorporated 0-5 knots. For the denominator, we also incorporated a term representing the time since progression squared.

The third and final step of the IPCW method involves the estimation of an HR for the outcome of interest – that is, the HR of death associated with being randomized to the trametinib group compared to the chemotherapy group. In line with Fewell *et al.* we use a pooled logistic regression for this final model, using baseline variables and the treatment arm indicator as covariates, and incorporating the time-varying, subject-specific stabilized weights.^10^ Again we used the rcsgen Stata command to generate restricted cubic splines for a time-dependent intercept. This pooled logistic regression is equivalent to a Cox proportional hazards model in cases where the hazard of the event is small in each time period. Because we split out dataset into daily observations this is true, and thus the pooled logistic model can be used and the odds ratio associated with trametinib can be interpreted as an HR.^10^

In our application of the IPCW method we attempted to include all covariates upon which baseline and time-dependent data were available, in an attempt to maximize the probability of satisfying the ‘no unmeasured confounders’ assumption. However, in some analyses models did not converge or implausible results were obtained and alternative analyses were run that excluded some covariates. To identify a suitable model we followed a stepwise procedure. First, we attempted to fit models including all covariates. If these did not converge, or results were implausible, we re-ran the analysis excluding information on the EQ-5D score. We deemed that this was appropriate because the more detailed data on quality of life as measured by the EORTC QLQ-30 was retained. If these models did not converge, or produced implausible results, we re-ran the analysis excluding information on the EORTC variables, but including information on the EQ-5D utility score. We deemed that this was appropriate because although the more detailed data on quality of life as measured by the EORTC QLQ-30 was excluded, EQ-5D data on quality of life was retained.

We followed this step-wise approach for the subgroup analyses of the first-line metastatic primary efficacy population. In this subgroup IPCW model convergence was more problematic: the logistic model for the denominator of the stabilized weight often did not converge and weights were extremely unstable (with maximum weights of > 1,000,000) when EORTC QLQ-30 data (either with or without additional information on the EQ-5D utility score) were included, hence we had to use models that excluded data on EORTC QLQ-30 but included EQ-5D scores. In some cases, even these did not produce plausible results.

We tested models fit with 0-5 knots incorporated within the restricted cubic splines estimated for our time-dependent intercept. We chose between these applications based upon Akaike information criterion (AIC) statistics in the final pooled logistic regression for the adjusted treatment effect. In our manuscript results are presented from the application that successfully converged and provided the lowest AIC statistic for the final pooled logistic model.

For the ‘all patients’ analysis the preferred IPCW model included all baseline and time-dependent covariates, except EQ-5D utility scores, and included 5 knots (therefore 4 splines) within the restricted cubic splines used for the time-dependent intercept. Our a priori preferred model, that included covariates for the EQ-5D utility score, produced unexpected results for the coefficient of the baseline EQ-5D utility score in the pooled logistic model for the denominator of the stabilized weight. In this model, the baseline EQ-5D utility score was associated with an odds ratio of 1,397.18, and the subsequently calculated stabilized weights had a maximum of 70.65 which, according to previous research, is likely to result in biased estimates of the treatment effect.^12,13^ The models that excluded EQ-5D covariates (but retained EORTC-QLQ 30 covariates, and thus retained a measure of health related quality of life) converged in a more satisfactory way, without any spurious coefficients, and the range of the stabilized weights was narrower – with a maximum of 18.75.

For the ‘first-line’ metastatic treatment subgroup analysis the preferred IPCW model included data on all baseline and time-dependent covariates in the analysis, except EORTC-QLQ 30 covariates, and included 5 knots (therefore 4 splines) within the restricted cubic splines used for the time-dependent intercept. Our a priori preferred model, that included covariates for EORTC and EQ-5D covariates, produced unexpected results for the coefficients of several covariates in the pooled logistic model for the denominator of the stabilized weight. For example, variables for receipt of prior immunotherapy, serious adverse events and baseline ECOG score all had odds ratios of > 90. The subsequently calculated stabilized weights had a maximum of 5,430.99 and it was therefore clear that this analysis was not stable. Models that excluded EQ-5D covariates (but retained EORTC-QLQ 30 covariates) also produced results that suggested that the application had not performed satisfactorily: for example, the odds ratio associated with the best response variable was 258,699.5 and subsequently calculated stabilized weights had a maximum of 665.22. Due to these issues, we focused upon the IPCW application that included EQ-5D covariates but excluded EORTC covariates, which converged in a more satisfactory way, without any spurious coefficients, and the range of the stabilized weights was narrower – with a maximum of 9.82.

Confidence intervals around the adjusted treatment effect for the IPCW were estimated incorporating robust standard errors, to allow for within-subject correlation caused by the weights.^10^

***Implementation of the 2-stage accelerated failure time model method***

Our application of the 2-stage estimation method involved 3 main steps. Step 1 involved constructing a dataset from which an estimate of the treatment effect received specifically by treatment switchers could be obtained. This dataset included only patients in the control group who had experienced disease progression, and included information on all covariates that was known at the time of disease progression. Where measurements were not available at the time of progression, the most recent measurement prior to progression was carried forward. For each patient included in this dataset, the time from disease progression until death or censoring was calculated.

Step 2 involved fitting a parametric accelerated failure time model to the dataset constructed in Step 1 to estimate the treatment effect on time to death from progression of switching onto trametinib. The following covariates were included in this model, along with a switching indicator:

- Baseline: Age (continuous variable)
- Sex (0 = Male; 1 = Female)
- Baseline: Receipt of prior chemotherapy for advanced or metastatic disease (0 = No; 1 = Yes)
- Baseline: Receipt of prior immunotherapy (0 = No; 1 = Yes)
- Baseline: ECOG performance status code (0 = ECOG of 0; 1 = ECOG of ≥ 1)
- Baseline: Lactate dehydrogenase level (0 = equal to or lower than upper normal limit; 1 = higher than upper normal limit)
- Baseline: Disease stage at screening (0 = Stage III, IVM1a or IVM1b; 1 = IVM1c)
- Baseline: Visceral disease at screening (0 = No; 1 = Yes)
- Baseline: Number of disease sites (0 = ≥ 3 sites; 1 = < 3 sites)
- Baseline: Sum of lesion diameters (continuous variable)
- Baseline: EQ-5D utility score (continuous variable)
- Baseline: EORTC quality of life 30 score (physical, role, emotional, cognitive, social, fatigue, nausea and vomiting, pain, dyspnea, insomnia, appetite loss, constipation, diarrhea, financial difficulties, global health status domains) (continuous variables)
- Time of progression: Time of progression (continuous variable)
- Time of progression: ECOG performance status code (0 = ECOG of 0; 1 = ECOG of ≥ 1)
- Time of progression: LDH (0 = equal to or lower than upper normal limit; 1 = higher than upper normal limit)
- Time of progression: Best response on study treatment (0 = Not evaluable; 1 = Progressive disease; 2 = Stable disease; 3 = Partial response; 4 = Complete response)
- Time of progression: Sum of lesion diameters (continuous variable)
- Time of progression: Presence of new lesions (0 = No; 1 = Yes)
- Time of progression: Serious adverse event experienced (0 = No; 1 = Yes)
- Time of progression: Serious adverse event current (0 = No; 1 = Yes)
- Time of progression: EQ-5D utility score (continuous variable)
- Time of progression: EORTC quality of life 30 score (physical, role, emotional, cognitive, social, fatigue, nausea and vomiting, pain, dyspnea, insomnia, appetite loss, constipation, diarrhea, financial difficulties, global health status domains) (continuous variable)

We attempted to fit Weibull and Generalized Gamma models, in order to determine whether the results were sensitive to the choice of accelerated failure time model. However, often models did not converge when all of the variables listed above were included, particularly in the first-line subgroup. In line with the approach taken in our IPCW analysis, our preference was for models that included all covariates upon which baseline and time-dependent data were available, in an attempt to maximize the probability of satisfying the ‘no unmeasured confounders’ assumption. When these full models did not converge we first attempted to fit models excluding EQ-5D utility score data, and if this failed we attempted to fit models excluding all EORTC QLQ-30 covariates (but retaining data on the EQ-5D utility score). We deemed that this was appropriate because both sets of covariates provide information on quality of life.

Step 3 involved using the treatment effect (in terms of an AF, $\mu_{B}$) determined by Step 2 to estimate counterfactual survival times for switchers by using equation (4). Recensoring was incorporated at this stage.

$U_{i}=T_{A_{i}}+\frac{T_{B_{i}}}{\mu_{B}}$ (4)

Where $T_{A_{i}}$ represents the time spent on control treatment, $T_{B_{i}}$ represents the time spent on the new intervention, and $\mu_{B}$ is the treatment effect (AF) in switching patients.

Finally, a Cox proportional hazards regression model, stratified for LDH and prior chemotherapy for advanced or metastatic disease, was used to estimate the treatment effect associated with being randomized to trametinib, comparing the trametinib failure times as observed in the METRIC trial and re-censored adjusted failure times for chemotherapy patients based on the estimate of $\mu_{B}$ produced by Step 2. To investigate the potential impact of recensoring, once potentially appropriate applications of the 2-stage method had been identified we re-ran these without incorporating recensoring.

As described above, 2 of the 64 primary efficacy population chemotherapy patients who switched onto trametinib did so before disease progression had been observed. To investigate the impact of this we ran 2 sets of 2-stage estimation analyses – in 1 set these patients were not included in the Step 1 dataset and so the treatment effect that they received was not incorporated within the estimation of the average treatment effect received by treatment switchers. Their survival times were then adjusted in Step 3 based upon the average treatment effect received by patients who switched treatments after disease progression. In the second set we assumed that disease progression was observed at the time of switching, and thus these patients were included within the Stage 1 dataset, and the treatment effect that they received was included in the estimation of the average treatment effect received by treatment switchers. Both approaches are prone to bias, because in the set 1 analyses we may under-adjust survival times in these 2 patients if these patients received a higher treatment effect due to receiving the treatment (apparently) before disease progression. On the other hand, the set 2 analyses may be biased because 2 patients are included in the estimation of the treatment switching treatment effect who had (apparently) not experienced disease progression and whom therefore are not at the same disease stage – the ‘secondary baseline’ is not equal for all patients. However, because only 2 patients are involved, resulting bias may be expected to be small.

For the ‘all patients’ analysis the preferred 2-stage accelerated failure time model application used a Weibull model that incorporated all covariates. Generalized Gamma models did not converge, even when EQ-5D or EORTC variables were excluded. It was assumed that the 2 patients who switched onto trametinib before disease progression had been observed actually had experienced disease progression at the time of switch (set 2 analysis) – this provided similar but marginally more conservative estimates of the adjusted treatment effect, compared to the set 1 analysis.

For the ‘first-line’ metastatic treatment subgroup the preferred 2-stage accelerated failure time model application used a Weibull model that incorporated all covariates, except EORTC covariates. Weibull and Generalized Gamma models that included all covariates did not converge. Similarly, models that excluded EQ-5D covariates but included EORTC covariates did not converge and the Generalized Gamma model that excluded EORTC covariates also failed to converge. Therefore, the Weibull model that included EQ-5D variables but not EORTC variables was the most complete model that successfully converged. It was assumed that the two patients who switched onto trametinib before disease progression had been observed had *not* experienced disease progression at the time of switch (set 1 analysis) – this provided similar but marginally more conservative estimates of the adjusted treatment effect, compared to the set 2 analysis.

To estimate confidence intervals around the adjusted treatment effect for the 2-stage estimation method we chose to retain the *P*-value from the ITT analysis – allowing consistency with the RPSFTM method.

**SUPPORTING REFERENCES**

1. Mark SD, Robins JM. A Method for the Analysis of Randomized Trials with Compliance Information - An Application to the Multiple Risk Factor Intervention Trial. *Controlled Clin Trials*. 1993;14:79-97.
2. Latimer NR, Abrams KR, Lambert PC, et al. Adjusting survival time estimates to account for treatment switching in randomised controlled trials – an economic evaluation context: Methods, limitations and recommendations. *Med Decis Making.* 2014;34:387-402.
3. White IR, Babiker AG, Walker S, et al. Randomization-based methods for correcting for treatment changes: Examples from the Concorde trial. *Stat Med.* 1999:18:2617-2634.
4. Yamaguchi T and Ohashi Y. Adjusting for differential proportions of second-line treatment in cancer clinical trials. Part I: Structural nested models and marginal structural models to test and estimate treatment arm effects. *Stat Med.* 2003;23:1991-2003.
5. Yamaguchi T, Ohashi Y. Adjusting for differential proportions of second-line treatment in cancer clinical trials. Part II: An application in a clinical trial of unresectable non-small-cell lung cancer. *Stat Med.* 2004;23:2005-2022.
6. Robins JM, Greenland S. Adjusting for Differential Rates of Prophylaxis Therapy for Pcp in High-Dose Versus Low-Dose Azt Treatment Arms in An Aids Randomized Trial. *J Am Stat Assoc.* 1994;89:737-749.
7. Mark SD, Robins JM. Estimating the Causal Effect of Smoking Cessation in the Presence of Confounding Factors using a Rank Preserving Structural Failure Time Model. *Stat Med.* 1993;12:1605-1628.
8. White IR, Walker S, Babiker A, et al. Strbee: Randomization-based efficacy estimator. *The Stata Journal.* 2002;2:140-150.
9. Robins JM and Tsiatis AA. Correcting for non-compliance in randomized trials using Rank Preserving Structural Failure Time Models. *Commun Stat Theory Methods.* 1999;20:2609-2631.
10. Fewell Z, Hernan MA, Wolfe F, et al. Controlling for time-dependent confounding using marginal structural models. *The Stata Journal.* 2004;4:402-420.
11. Hernan MA, Brumback B, Robins JM. Marginal structural models to estimate the joint causal effect of nonrandomized treatments. *Journal of the American Statistical Association.* 2001;96:440-448.
12. Latimer N, Abrams K, Lambert P, et al. Adjusting for treatment switching in randomised controlled trials – a simulation study. University of Sheffield Health Economics and Decision Science Discussion Paper No.13/06. 12-7-2013
13. Latimer N, Abrams K, Lambert P, et al. Assessing methods for dealing with treatment switching in clinical trials: A follow-up simulation study. University of Sheffield Health Economics and Decision Science Discussion Paper No.14/01. 5-3-2014
